# Meetings of Societies

**Published:** 1907-09

**Authors:** 


### MEETINGS OF SOCIETIES.

### Bristol Medico-Chirurgical Society.

*June* 12*th*, 1907, *at Weston-super-Mare.*

Mr. James Taylor, President, in the Chair.

Dr. Charles and Prof. Walker Hall showed specimens from a case of **Carcinoma of the Stomach.**

Dr. H. Stanley Ballance showed a female patient upon whom **Estlander's Operation for Empyema** had been successfully performed. The following is the history of the case in brief :—Mrs. B., æt. 45, first became ill in October, 1901. On July 27th, 1902, the left side was found to be almost motionless during respiration ; there were signs of a large amount of fluid ; the temperature was 103°. Part of the 9th rib in the posterior axillary line was removed, and a good deal of pus evacuated. One month later part of the 8th rib was removed, and a second and smaller pus-containing cavity opened. On February 6th, 1904, a probe could be passed upwards to the level of the 1st rib ; parts of the 5th, 6th, 7th, 8th and 9th ribs, with intervening soft parts and parietal pleura. were removed. On February 27th, 1904, parts of the 2nd, 3rd and 4th ribs were similarly removed. Eleven days later symptoms of acute pericarditis appeared, but passed off rapidly on administering antistreptococcus serum. The patient improved until the following July, and then for 3½ months was melancholic. The wound is now healed, and the patient living her ordinary life.

Dr. Roxburgh read a paper on a case of **Banti's Disease with Complications.** The patient (female), aged 48, presented symptoms of extreme anæmia, slight dilatation of the heart, enlargement of spleen and liver, a hectic temperature and deficient air entry, with crepitations in the left upper and right middle pulmonary lobes. These conditions had begun three months before admission (though dyspnœa on exertion had been observed two years earlier). On admission the liver reached 2½ inches below the right costal margin, and the spleen, which was peculiarly hard to the sense of touch, projected two finger breadths below the left costal edge. The blood count was erythrocytes, 2,320,000; leucocytes, 6,316; no poikilocytes; hæmoglobin decidedly diminished. In twenty-three days these figures had fallen respectively to 1,111,000 and 4,900; general pigmentation of the skin had become marked; and pulmonary signs more pronounced, including whispering pectoriloquy at the left apex. No tubercle bacilli were found in the scanty sputum. The spleen had increased in size, and the liver extended seven inches below the costal margin. Five weeks after admission, the patient died, and the autopsy showed, put very shortly:—Heart: Fatty degeneration. Lungs: Right firmly adherent in most of its extent to the thoracic wall and diaphragm; both lungs in a state of fibrous cirrhosis, the result of chronic interstitial pneumonia; no bronchiectasis, no caseation or tubercles. Bronchial glands large, hard, and black, as in anthracosis, but not tubercular. Spleen: 7.5 inches in length, tough and hard, with thickened capsule, on section fibrous; Malpighian bodies indefinite. Liver: Peculiarly formed, the right lobe extending almost to the iliac crest; the whole organ very firm and tense, in a state of fine cirrhosis with fatty degeneration. Kidneys and supra-renals normal. No tubercle of mesenteric glands, but the mesentery dotted with small black spots, probably former petechiæ. Microscopically, the extreme fibrosis of spleen, liver and lymphatic glands was confirmed. These irritative changes and the train of symptoms leading directly to death, pointed to some such chronic intoxication as that which presumably occurs in Banti's disease; but in the course of that syndrome the splenomegaly seems to precede the anæmia and enlargement of liver, sometimes by several years. In the absence of a complete history it was impossible to say whether such had been the case here. The chronic interstitial changes in the lungs may have been due directly (or indirectly, following a chronic interstitial inflammation) to the same toxin.

Dr. Armstrong's paper at last year's Toronto meeting of the British Medical Association gave an excellent *résumé* of the subject and a complete list of all the cases of Banti's disease up to that date treated by excision of the spleen. This list comprised 32 cases, with 23 recoveries, *i.e.* 72%. Dr. Armstrong concluded (1) that the disease is essentially progressive ; (2) that no treatment other than splenectomy is permanently effective in removing the anæmia and hindering the hepatic cirrhosis ; and (3) that even in advanced cases the operation is followed by immediate improvement, which often has a considerable amount of permanency. He laid special stress on the dilatation of the splenic vein, and even suggested that a primary endophlebitis of that vessel might set up the disease by causing backward pressure, injury to the vitality of the spleen pulp, and consequent production of an enzyme, which acted as a toxin to the liver. This was as yet purely theoretical, and the primary cause of the splenomegaly still remained obscure.—Dr. Roxburgh's paper was criticised by Dr. EDGEWORTH and Dr. MICHELL CLARKE. The former doubted whether the case properly should be classed as Banti's disease. The latter admitted the complexity of the case, and the great difficulty of its diagnosis.—Dr. ROXBURGH, in reply, affirmed his belief in the splenic origin of the symptom complex, while the pulmonary cirrhosis was probably an after effect, like the Hanot's cirrhosis of the liver, which was so constant.

Dr. CROUCH read a paper on a suggested **Treatment for Functional Aphonia.** (For this paper see p. 214.)

Dr. MICHELL CLARKE read a paper on the **Treatment of Graves's Disease.** (Vide p. 201.)

J. LACY FIRTH.

H. F. MOLE, *Hon. Sec.*

---

